# Antibody-drug conjugates in solid tumors; new strategy for cancer therapy

**DOI:** 10.1093/jjco/hyae054

**Published:** 2024-05-04

**Authors:** Toshiaki Takakura, Toshio Shimizu, Nobuyuki Yamamoto

**Affiliations:** Department of Pulmonary Medicine and Medical Oncology, Wakayama Medical University Faculty of Medicine, 811-1 Kimiidera, Wakayama, Wakayama 641-8510, Japan; Department of Pulmonary Medicine and Medical Oncology, Wakayama Medical University Faculty of Medicine, 811-1 Kimiidera, Wakayama, Wakayama 641-8510, Japan; Department of Pulmonary Medicine and Medical Oncology, Wakayama Medical University Faculty of Medicine, 811-1 Kimiidera, Wakayama, Wakayama 641-8510, Japan

**Keywords:** antibody-drug conjugate, solid tumors, targeted cancer therapy

## Abstract

Antibody-drug conjugates (ADCs) have emerged as a novel class of anticancer treatment. ADCs are composed of three parts: a monoclonal antibody, a linker and a payload. A monoclonal antibody binds to the specific antigen present at the cancer cells, allowing selective delivery of the cytotoxic agents to the tumor site. Several ADCs are approved by the US Food and Drug Administration for the treatment of hematologic cancers and solid tumors with clinically meaningful survival benefit. However, the development of ADCs faces a lot of challenges and there is a need to get better understanding of ADCs in order to improve patient outcomes. Here, we briefly discuss the structure and mechanism of ADCs, as well as the clinical data of current approved ADCs in solid tumors.

## Teaser

This article reviews recent trends of ADCs’ drug development against solid tumors. The ability to deliver payloads selectively to targeting cancer cells potentially enable strengthen efficacy and improve safety profiles.

## Introduction

**Figure 1:**
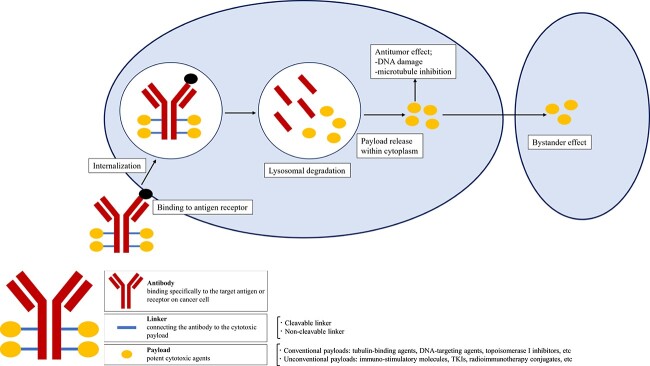
Structure of antibody-drug conjugates (ADCs). ADCs have three components: a monoclonal antibody that binds to an antigen or receptor on cancer cells, a linker that connects the antibody and payload, and a payload that is a potent cytotoxic agent. ADCs bind to target antigens on the surface of cancer cell followed by internalisation. After lysosomal degradation, payloads are released into the cytoplasm and induces cell death via DNA damage or microtubule inhibition. Moreover, payloads pass through the cell membrane and show antitumor effect on surrounding cancer cells, called bystander effect.

The current systemic therapeutic agents against solid tumors comprise chiefly chemotherapy, targeted therapy, immune checkpoint inhibitors (ICIs). Although these therapies have improved outcomes, better ways of treatment are expected. Antibody-drug conjugates (ADCs) are rapidly emerging class of therapeutic agents combining cytotoxic drugs and targeted antibodies via a chemical linker (Fig. 1). The concept of targeted chemotherapy was first proposed by a German scientist, Paul Ehrlich 100 years ago, known as ‘magic bullet’.

The US Food and Drug Administration (FDA) firstly approved Gemtuzumab ozogamicin (Mylotarg®) in 2000 for the treatment of adults with CD33-positive acute myeloid leukemia (1). As for solid tumors, trastuzumab emtansine (T-DM1) was approved by FDA in 2013 for the treatment of metastatic breast cancer. As of 1 November 2023, the FDA has approved 13 ADCs for solid or hematologic cancers (6 for solid tumors) (Table 1, 2). In this review, we aim to provide a brief overview of the ADCs for solid tumors, their structure and mechanism of action, available clinical trial data.

**Table 1:** FDA-approved antibody-drug conjugates (ADCs) for solid tumors

ADCs	Target	Linker	Payload	DAR	Cancer type	First approved date by FDA	First approved date in Japan
Trastuzumab emtansine (T-DM1)	HER2 (overexpression/gene expression)	Non-Cleavable	Maytansine (DM1)	3.5	HER2+ BC	22 Feb 2013	20 Sep 2013
Enfortumab vedotin (EV)	Nectin-4	Cleavable	MMAE	4	Urothelial cancer	18 Dec 2019	27 Sep 2021
Trastuzumab deruxtecan (T-DXd)	HER2 (overexpression/gene expression)	Cleavable	Deruxtecan	8	HER2+/HER2 low BC, HER2+ GC/GOJ adenocarcinoma	20 Dec 2019	25 Mar 2020
	HER2 (gene mutation)				HER2+ NSCLC		
Sacituzumab govitecan (SG)	TROP-2	Cleavable	SN-38	7.6	TNBC, urothelial cancer	22Apr 2020	-
Tisotumab vedotin	Tissue factor	Cleavable	MMAE	4	Cervical cancer	20 Sep 2021	-
Mirvetuximab soravtansine	FR	Cleavable	DM4	3–4	Ovarian cancer	14 Nov 2022	-

**Table 2:** Selected clinical trial data

	Trial	Populations	Intervention	Overall response rate (ORR) (95% CI)	Median progression-free survival (mPFS) (95% CI)	Median overall survival in months (mOS) (95% CI)	Ref
Trastuzumab emtansine (T-DM1)	EMILIA (Phase3)	HER2+ BC (Pretreated with trastuzumab +taxane)	T-DM1 vs capecitabine +rapatinib	43.6% (38.6–48.6) vs 30.8% (26.3–35.7)	9.6 m vs 6.4 m HR 0.65 (0.55–0.77)	30.9 m vs 25.1 m HR 0.68 (0.55–0.85)	8
	KATHERINE (Phase3)	early HER2+ BC (residual invasive disease after neoadjuvant therapy)	T-DM1 for 14 cycles vs trastuzumab for 14 cycles		(3 years invasive disease free survival) 88.3% vs 77.0% HR 0.50 (0.39–0.64)		9
Enfortumab vedotin (EV)	EV-301 (Phase3)	Urothelial carcinoma (treated with platinum and ICI)	EV vs chemotherapy	40.6% (34.9–46.5) vs 17.9% (13.7–22.8)	5.6 m vs 3.7 m HR 0.62 (0.51–0.75)	12.9 m vs 8.9 m HR 0.70 (0.56–0.89)	10
Trastuzumab Deruxtecan (T-DXd)	DESTINY-Breast01 (Phase2)	HER2+ BC (pretreated with T-DM1)	T-DXd (5.4 mg/kg)	60.9% (53.4–68.0)	16.4 m (12.7-NE)	NR	11
	DESTINY-Breast03 (Phase3)	HER2+ BC (pretreated with trastuzumab)	T-DXd (5.4 mg/kg) vs T-DM1	79% (73.1–83.4) Vs 35% (29.2–41.1)	28.8 m vs 6.8 m HR 0.33 (0.26–0.43)	NR (40.5-NE) vs NR (34.0-NE) HR 0.64 (0.47–0.87)	12
	DESTINY-Breast04 (Phase3)	Pretreated HER2 low BC	T-DXd (5.4 mg/kg) vs chemotherapy	52.3% (47.1–57.4) Vs 16.3% (11.3–22.5)	9.9 m vs 5.1 m HR 0.50 (0.40–0.63)	23.4 m vs 16.8 m HR 0.64 (0.49–0.84)	13
	DESTINY-Gastric01 (Phase 2)	Gastric cancer (pretreated with trastuzumab)	T-DXd (6.4 mg/kg) vs chemotherapy	43% (34–52) vs 12% (5.0–24.0)	5.6 m vs 3.5 m HR 0.47 (0.31–0.71)	12.5 m vs 8,4 m HR 0.59 (0.39–0.88)	14
	DESTINY-Lung01 (Phase 2)	Pretreated HER2 mutation+ NSCLC	T-DXd (6.4 mg/kg)	55% (44.0–65.0)	8.2 m (6.0–11.9)	17.8 m (13.8–22.1)	15
	DESTINY-Lung02 (Phase2)	Pretreated HER2 mutation+ NSCLC	T-DXd (5.4 mg/kg) vs T-DXd (6.4 mg/kg)	49.0% (39.0–59.1) vs 56.0% (41.3–70.0)	9.9 m (7.4-NE) vs 15.4 m (8.3-NE)	19.5 m (13.6-NE) vs NE (12.1-NE)	16
Sacituzumab govitecan (SG)	IMMU-132-01 (Phase1/2)	Solid tumors	SG 8, 10,m12 mg/kg (only 10 mg/kg group was analyzed)				17
		TNBC cohort		33.3% (24.6–43.1)	5.6 m (4.8–6.6)	13.0 m (11.2–14.0)	17
		HR+ BC cohort		31.5% (19.5–45.6)	5.5 m (3.6–7.6)	12.0 m (9.0–18.2)	17
		NSCLC cohort		16.7% (7.9–29.3)	4.4 m (2.5–5.4)	7.3 m (5.6–14.6)	17
	ASCENT (Phase3)	Pretreated TNBC	SG (10 mg/kg) vs chemotherapy	35% vs 5% (not reported)	5.6 m vs 1.7 m HR 0.41 (0.32–0.52)	12.1 m vs 6.7 m HR 0.48 (0.38–0.59)	18
	Tropics-02 (Phase3)	Pretreated HR+/HER2-BC	SG (10 mg/kg) vs chemotherapy	21% vs 14% Odds Ratio 1.63 (1.03–2.56)	5.5 m vs 4.0 m HR 0.66 (0.53–0.83)	14.4 m vs 11.2 m HR 0.79 (0.65–0.96)	19
Tisotumab vedotin	innovaTV 204/GOG-3023/ENGOT-cx6 (Phase2)	Pretreated cervical cancer	Tisotumab vedotin (2.0 mg/kg)	24% (16.0–33.0)			20
Mirvetuximab soravtansine (MIRV)	SORAYA (Phase2)	Platinum resistance epithelial ovarian cancer	MIRV (6 mg/kg)	32.4% (23.6–42.2)	4.3 m (3.7–5.2)	13.8 m (12.0-NR)	21

## Structure and mechanism

### Antibody

Monoclonal antibodies are the components that allow the ADCs to bind specifically to the target antigen or receptor on cancer cell. The antibodies for ADCs require an adequate binding affinity, efficient internalisation, low immunogenicity and a long half-life. Murine antibodies were often used in the early days of ADCs development, but their high immunogenicity made them likely to cause serious side effects. Currently, development is increasingly used chimeric, humanised and fully humanised antibodies with low immunogenicity. In the five types of Immunoglobulin (M, A, D, E, G), Immunoglobulin G (IgG) is the most commonly used in ADCs. IgGs are classified into IgG1–4, but IgG3 is usually not used because of its short half-life, and IgG1 is widely used due to its immunogenic functions such as antibody-dependent cellular cytotoxicity (ADCC), antibody-dependent cell-mediated phagocytosis (ADCP) and complement-dependent cytotoxicity (CDC) (2).

### Payload

Potent cytotoxic agents are the ‘payloads’ of ADCs. Many of the cytotoxic agents in ADCs are very low IC50 (the drug-concentration to inhibit the growth of 50% of cells), so that they cannot be used as drugs on their own because they exhibit strong toxicity. The payloads chiefly classified into tubulin-binding agents (auristatins or maytansinoids), DNA-targeting agents (calicheamicins, duocarmycin), topoisomerase I inhibitors (deruxctecan, SN-38). In these cytotoxic agents, auristatins are the largest family of payloads so far, due to their favorable biochemical characteristics. Auristatins include two types of derivatives: monomethyl auristatin E (MMAE) and monomethyl auristatin F (MMAF). While the MMAF shows low membrane permeability, the MMAE has favorable permeability to cell membrane and can diffuse into nearby cancer cells and kill them, called the bystander effects. Currently, in approved ADCs for solid tumors, MMAE is loaded in enfortumab vedotin (EV) and tisotumab vedotin (3).

Furthermore, several types of alternative payloads are under development, such as radionucleotides, immune modulators, tyrosine kinase inhibitors and dual-distinct payloads. Moreover, the conjugation of payloads to bispecific antibodies is another strategy (4). The drug-antibody ratio (DAR) is the amount of payloads loaded to the antibody and is a critical factor to determine the efficacy of the ADCs. ADCs with higher DAR values increase potency in vitro but are usually less favorable in vivo due to the unstable structure and low binding capacity, resulting in faster plasma clearance. Therefore, in most of ADCs, their DAR values are in the range of 2–4. However, several ADCs have emerged with favorable pharmacokinetics profiles even in DAR values of around 8, such as trastuzumab deruxtecan (T-DXd) and sacituzumab govitecan (SG) (5,6).

### Linker

Linkers connect the antibody to the cytotoxic payload and define several characteristics of ADCs. Ideal linkers should ensure ADCs stability in the bloodstream but release payloads at the tumor site. The conjugation site has a significant impact on the stability and pharmacokinetics of ADCs. Site-specific binding produces homogeneous ADCs whose properties can be tailored to maximise therapeutic range (7).

Linkers can be categorised into cleavable and non-cleavable. Cleavable linkers release the payload based on some factors of cancer cell such as pH, reducing agents and lysosomal protease. While cleavable linkers have the advantage of providing a bystander effect, they have the disadvantage of increased toxicity in normal tissues. Conversely, non-cleavable linkers are more stable in plasm, so that they release payload only after complete lysosomal degradation of the antibody and limit premature release of payload. Whereas these features of non-cleavable linkers offer less toxicity, bystander effect is not expected because of the extremely low cell membrane permeability. Among the 13 approved ADCs, cleavable linkers are the most common (11 of 13) and non-cleavable linkers are only two (trastuzumab emtansine, belantamab mafodotin).

### Mechanism of action

The action of ADCs begins with the recognition and binding of target antigens on the surface of cancer cell. After that, the ADCs are internalised through endocytosis with formation of early endosomes. The early endosomes mature into late endosomes, which then fuse with lysosomes. Whereas ADCs with cleavable linkers release their payload depending on the localisation of enzymes and other factors, the ADCs with non-cleavable linkers release their payloads when antibodies are degraded in lysosomes. The payloads are released into the cytosol and induces cell death via DNA damage or microtubule inhibition. In addition, after the payloads are released intracellularly, they may pass through the cell membrane and exert an antitumor effect on surrounding cancer cells, referred to as the bystander effect. The bystander effect is important because tumors are heterogeneous populations of cancer cells and target antigens are not expressed by all cancer cells. This bystander effect may explain the efficacy of T-DXd in patients with ‘HER2-low’ breast cancer. Furthermore, the ADCs display anticancer effect through immunogenic reactions including ADCC, ADCP and CDC.

## ADCs for solid tumors

### Trastuzumab emtansine (T-DM1)

T-DM1 is the first ADC approved for solid tumors (8). It combines the anti-HER2 IgG1 monoclonal antibody to the cytotoxic payload maytansine (DM1), using a non-cleavable linker. FDA approved T-DM1 for the treatment of HER2-positive metastatic breast cancer (BC) based on the results of EMILIA trial, a global phase III trial. The overall response rate (ORR) was 43.6 vs 30.8%, median progression free survival (mPFS) was 9.6 m (HR 0.65, 95%CI 0.55–0.77), median overall survival (mOS) was 30.9 m (HR 0.68, 95% CI 0.55–0.85). The most frequent grade ≥ 3 treatment-emergent adverse events (TEAEs) were thrombocytopenia (12.9%) and elevated serum concentrations of aspartate aminotransferase (4.3%) and alanine aminotransferase (2.9%).

In addition, it is used for an adjuvant therapy for residual disease in early-stage HER2-positive BC after neoadjuvant therapy, on the basis of KATHERINE trial (9). In this phase III trial, T-DM1 (for 14 cycles) was compared with trastuzumab for patients with HER2-positive early breast cancer who were found to have residual invasive disease at surgery after neoadjuvant therapy including a trastuzumab and a taxane (with or without anthracycline). 3 years invasive disease free survival was 88.3% (HR 0.50, 95%CI 0.39–0.64). Grade ≥ 3 TEAEs were reported that a thrombocytopenia (5.7%) and hypertension (2.0%). Assigned therapy were completed in 71.4 (T-DM1 group) and 81.0% of patients (trastuzumab group). T-DM1 was discontinued due to thrombocytopenia (4.2%), elevated blood bilirubin level (2.6%), elevated aspartate aminotransferase level (1.6%), elevated alanine aminotransferase level (1.5%), peripheral sensory neuropathy (1.5%) and decreased ejection fraction (1.2%).

### Enfortumab vedotin

Nectin-4 is a cell-adhesion molecule that may associated with cancer cell growth and proliferation and overexpressed in urothelial cancer and breast cancer. EV is an ADC consisting of a fully human monoclonal nectin-4 antibody and monomethyl auristatin E (MMAE) (10). A phase III trial EV-301 was conducted to evaluated EV as compared with investigator’s choice chemotherapy (docetaxel, paclitaxel or vinflunine) in patients with locally advanced or metastatic urothelial cancer who had previously treated with a platinum-based chemotherapy and PD-1/PD-L1 inhibitor. The mPFS was 5.6 months (HR 0.62, 95% CI 0.51–0.75) and the mOS was 12.9 months (HR 0.70, 95% CI 0.56–0.89). The most common grade ≥ 3 treatment-related adverse events (TRAEs) were maculopapular rash (7.4%), fatigue (6.4%) and decreased neutrophil count (6.1%).

### Trastuzumab deruxtecan

T-DXd consists of a humanised HER2 antibody (trastuzumab) and topoisomerase I inhibitor (deruxtecan) (11). The FDA approved T-DXd for the treatment of pretreated HER2-positive metastatic BC based on the data of a phase II trial, DESTINY-Breast01. Then, in DESTINY-Breast03, a phase III trial comparing T-DXd with T-DM1 in metastatic HER2-positive BC, T- DXd demonstrated clinical advantage (12). The mPFS was 28.8 months (HR 0.33, 95% CI 0.26–0.43) and mOS was NR (HR 0.64, 95% CI 0.47–0.87). The most common grade ≥ 3 TEAEs where neutrophil count decreased (16%), anemia (9%), platelet count decreased (8%), nausea (7%). Drug-related interstitial lung disease (ILD) or pneumonitis occurred in 15% of patients, including grade 1 (4%), grade 2 (10%), grade 3 (<1.0%) and grade ≥ 4 (0%).

Furthermore, DESTINY-Breast04, a phase III trial, was conducted to investigate the efficacy of T-DXd in the treatment of HER2-low metastatic BC (13). This study revealed a meaningful improvement in mPFS (HR 0.50, 95% CI 0.40–0.63) and mOS (HR 0.64, 95% CI, 0.49–0.84). The most frequent grade ≥ 3 AEs were neutropenia (13.7%), anemia (8.1%) and fatigue (7.5%). Drug-related ILD or pneumonitis occurred in 12.1% of patients, with grade 1 (3.5%), grade 2 (3.5%), grade 3 (1.3%) and grade 5 (0.8%).

In DESTINY-Gastric01, randomised phase II trial, T-DXd showed significant response as compared with chemotherapy in HER2-positive advanced gastric or gastroesophageal junction cancer patients who have previously treated with trastuzumab (14). The PFS was 5.6 months (HR 0.47, 95% CI 0.31–0.71) and the mOS was 12.5 months (HR 0.59, 95% CI 0.39–0.88). The most common grade ≥ 3 AEs were with decreased neutrophil count (51%), anemia (38%), a decreased white-cell count (21%), and decreased appetite (17%). A total of 12 patients (10%) had drug-related ILD or pneumonitis, with three events of grade 1, six events of grade 2, two events of grade 3, one event of grade 4 and no grade 5 events.

In phase II, DESTINY-Lung01 trial evaluated the efficacy of T-DXd (6.4 mg/kg) in the treatment of pretreated metastatic HER2-mutant NSCLC patients (15). The ORR was 55% (95% CI, 44–65), the mPFS was 8.2 months (95% CI, 6.0–11.9) and the mOS was 17.8 months (95% CI, 13.8–22.1). Grade ≥ 3 TRAEs occurred in 46% of patients, including neutropenia (19%) and anemia (10%). Drug-related ILD or pneumonitis were reported in 24 patients (26%), with 3 events of grade 1, 15 events of grade 2, 4 events of grade 3 and two events of grade 5. The median time to the onset of ILD was 141 days (range, 14–462).

Additionally, the clinical benefit of T-DXd (5.4 mg/kg) compared with T-DXd (6.4 mg/kg) was reported in phase II DESTINY-Lung02 trial (16). Both doses of T-DXd showed clinical activity; however, the safety profile was tolerable favoring T-DXd 5.4 mg/kg. Grade ≥ 3 drug-related TEAEs occurred in 38.6 (in 5.4 mg/kg group) and 58.0% patients (in 6.4 mg/kg group). Drug-related ILD were 12.9 and 28.0% of patients (grade ≥ 3 were 2.0% in both group), respectively.

### Sacituzumab govitecan

TROP2 (trophoblast antigen 2) is a transmembrane glycoprotein coded by the gene TACSTD2, which primarily acts as intracellular calcium signal transducer (17). While TROP2 is overexpressed in many human epithelial tissues, the expression increases in tumor tissue compared to normal tissues and has been associated with poor prognosis. This overexpression occurs in various tumor types including breast cancer, NSCLC, colon cancer, esophaegeal squamous cancer, thyroid cancer and hepatobiliary cancer.

SG, formerly IMMU-132, is composed of a humanised TROP2 antibody and the active metabolite of the topoisomerase I inhibitor irinotecan (SN-38).

ASCENT trial, a phase III trial comparing SG with a physician’s choice chemotherapy (eribulin, vinorelbine, capecitabine or gemcitabine) in TNBC patients, revealed a survival benefit of SG (18). The mPFS was 5.6 months (HR 0.41, 95% CI 0.32–0.52) and the mOS was 12.1 months (HR 0.48, 95% CI, 0.38–0.59). The most common grade ≥ 3 AEs were neutropenia (51%), leukopenia (10%), diarrhea (10%), anemia (8%) and febrile neutropenia (6%).

In the Tropics-02 study, a randomised phase III trial, SG was compared with physician’s choice chemotherapy (eribulin, vinorelbine, capecitabine or gemcitabine) in the HR+/HER2– breast cancer patients with endocrine-resistant, chemotherapy-treated (19). The mPFS was 5.5 months (HR 0.66, 95% CI 0.53–0.83) and the mOS was14.4 months (HR 0.79, 95% CI, 0.65–0.96). The most frequent grade ≥ 3 TEAEs were neutropenia (51%) and diarrhea (10%).

### Tisotumab vedotin

Tisotumab vedotin is an ADC consisting of a monoclonal antibody against tissue factor (TF) and MMAE (20). TF is a transmembrane glycoprotein physiologically expressed on fibroblasts and subendothelial cells and serves as an initiator of the extrinsic coagulation cascade. Conversely, TF is frequently overexpressed on several solid tumors, including cervical cancer and related to tumor growth, metastasis and angiogenesis.

A phase II trial, innovaTV 204/GOG-3023/ENGOT-cx6, reported that the sclinical activity of Tisotumab vedotin in patients with pretreated recurrent or metastatic cervical cancer. The confirmed ORR was 24% (95% CI, 16.0–33.0), including 7% of CR and 17% of PR. The most common grade ≥ 3 TRAEs included neutropenia (3%), fatigue (2%), ulcerative keratitis (2%) and peripheral neuropathies (2% each with sensory, motor, sensorimotor and neuropathy peripheral).

### Mirvetuximab soravtansine

Mirvetuximab soravtansine (MIRV) is an ADC with folate receptor an (Fra) antibody conjugated with the maytansinoid (DM4) as payload (21). In the SORAYA study, a single arm, phase II study investigated a clinical activity of MIRV in patients with FRa positive, platinum resistant epithelial ovarian cancer. The ORR was 32.4% (95% CI, 23.6–42.2), the mPFS was 4.3 months (95% CI, 3.7–5.2) and the mOS was 13.8 months (95% CI, 12.0-NR). The most common grade ≥ 3 TRAEs were blurred vision (6%), keratopathy (9%), dry eye (2%), diarrhea (2%) and neutropenia (2%).

### Datopotamab deruxtecan

Datopotamab deruxtecan (Dato-DXd) is a novel ADC composed of humanised anti TROP2 IgG1 monoclonal antibody, a cleavable linker, a topoisomerase I inhibitor payload and has a DAR of 4 (22). Although both of Dato-DXd and SG are the TROP2 ADCs, the two ADCs are different in some points. Whereas 90% of the payload of SG is released in 3 days, just 5% of the payload of Dato-DXd is released after 21 days. Because of this pharmacokinetic feature, Dato-DXd is administered every 3 weeks in contrast to SG dosed on Days 1 and 8.

TROPION-Pan Tumor01, a phase I basket trial, is investigating the safety and antitumor activity of Dato-DXd at dose levels of 4–8 mg/kg in unresectable advanced solid tumors. In patients treated at 6 mg/kg of NSCLC cohort, the ORR was 26% (95% CI, 14.6–40.3), the mPFS was 6.9 months (95% CI, 2.7–8.8) and the mOS was 11.4 months (95% CI, 7.1–20.6). The most frequent grade ≥ 3 TEAEs were pneumonia, anemia and lymphocytopenia. The potential ILD occurred in 28 patients across expansion doses (13 ≥ grade 3, 15 ≤ grade2).

Furthermore, some phase III trials of Dato-DXd are ongoing. TROPION-Lung 01, a phase III study evaluating Dato-DXd as compared with docetaxel (DTX) in pretreated NSCLC patients, demonstrated greater efficacy with manageable safety profile (23). The confirmed ORR was 26.4 (Dato-DXd) and 12.8% (DTX), the median duration of response (DOR) was 7.1 and 5.6 months, and the mPFS in non-squamous histology subgroup was 5.6 and 3.7 months (HR 0.63, 95% CI 0.51–0.78). The most common TRAEs in Dato-DXd group were stomatitis (49.2%, mostly grade 1–2) and nausea (37%). Drug-related ILD grade ≥ 3 occurred in 3.4% in Dato-DXd group and 1.4% in DTX group.

The combination therapies of Dato-DXd with other anticancer agents for NSCLC patients are investigated in TROPION-Lung07 (ClinicalTrials.gov identifier: NCT05555732), TROPION-Lung08 (ClinicalTrials.gov identifier: NCT05215340), AVANZAR (ClinicalTrials.gov identifier: NCT05687266). TROPION-Lung07, a phase III trial, is evaluating Dato-DXd plus pembrolizumab with or without a platinum agent in NSCLC with a PD-L1 tumor proportion score (TPS) of <50%.

TROPION-Lung08 is a phase III trial to evaluate Dato-DXd plus pembrolizumab comparing with pembrolizumab as first-line therapy for patients with a PD-L1 TPS of ≥50%. AVANZAR, a phase III study, examined the survival benefit of Dato-DXd plus durvalumab plus carboplatin against pembrolizumab plus platinum-based chemotherapy as first-line treatment.

In phase III TROPION-Breast01 trial, Dato-DXd was investigated in hormone (HR) positive/HER2 negative BC patients priory treated with endocrine therapy and cytotoxic chemotherapy. Dato-DXd showed clinical benefit (24). The confirmed response was 36.4% (Dato-DXd) and 22.9% (investigator’s choice of chemotherapy), and the mPFS was 6.9 months and 4.9 months (HR 0.63, 95% CI 0.52–0.76). OS data were not mature. The grade ≥ 3 TRAEs were less likely in Dato-DXd group.

### Patritumab deruxtecan (HER3-DXd)

Human epidermal growth factor receptor 3 (HER3) is a tyrosine kinase receptor that expressed in various solid tumors, including breast cancer and NSCLC (25,26). It is reported that high expression of HER3 is associated with poor prognoses and resistance mechanism of EGFR TKI.

HER3-DXd is a novel ADC composed of a humanised HER3 antibody and a topoisomerase I inhibitor payload. It has a high DAR value of 8. HERTHENA-Lung01 is a phase II trial to evaluate the efficacy and safety of HER3-DXd in EGFR mutated NSCLC patients previously treated with EGFR TKI and platinum-based chemotherapy (27). In this study, HER3-DXd demonstrated significant efficacy. The confirmed ORR was 29.8% (95CI, 23.9–36.2), the mPFS was 5.5 months (95% CI, 5.1–5.9) and the mOS was 11.9 months (95% CI, 11.2–13.1). Grade ≥ 3 TEAEs occurred in 64.9% of patients, including thrombocytopenia (20.9%), neutropenia (19.1%), anemia (14%) and leukopenia (10%). A phase III trial in EGFR mutated NSCLC patients pretreated with EGFR TKI is ongoing (HERTHENA-Lung02; ClinicalTrials.gov identifier: NCT05338970).

The U31402-A-J101 study is a phase I/II trial to assess the maximum tolerated dose (in the dose-escalation part) and the safety and efficacy (in the dose expansion part) of HER3-DXd in patients with previously treated HER3 expressing BC (28). In patients with HR+/HER2- BC, the confirmed ORR was 30.1% (95% CI, 21.8–39.4), the mPFS was 7.4 months (95% CI, 4.7–8.4) and the mOS was 14.6 months (95% CI, 11.3–19.5). In patients with TNBC, the confirmed ORR was 22.6% (95% CI, 12.3–36.2), the mPFS was 5.5 months (95% CI, 3.9–6.8) and the mOS was 14.6 months (95% CI, 11.2–17.2). In patients with HER2+ BC, the confirmed ORR was 42.9% (95% CI, 17.1–71.1), mPFS was 11.0 months (95% CI, 4.4–16.4), mOS was 19.5 months (95% CI, 12.2- NE). Grade ≥ 3 TEAEs occurred in 71.4% of all patients: 64.6 (4.8 mg/kg group) and 81.6% of patients (6.4 mg/kg group). In all patients, the most common grade ≥ 3 TEAEs were decreased neutrophil count (39.5%), decreased platelet count (30.8%), anemia (18.6%) and decreased white blood cell count (18.1%). Several studies to evaluate HER3-DXd are ongoing, such as ICARUS-Breast (ClinicalTrials.gov identifier: NCT04965766), SOLTI TOT-HER3 (ClinicalTrials.gov identifier: NCT04610528) and SOLTI-2103 VALENTINE (ClinicalTrials.gov identifier: NCT05569811).

## Future perspective

The development of ADCs has the following directions; stabilisation in vivo (increase hydrophilicity, site specific conjugation), enhanced efficacy (increase DAR, dual payload, bystander effect, combination therapy) and reduction of toxicity (tumor specific antigen, tumor site specific payload release, dose-optimisation). Improvements in antibodies, linkers, payloads and conjugation are underway to achieve these goals.

### Efficacy enhancement

In general, increasing hydrophilicity of ADCs result in improving stability in circulating plasma and anticancer effect. One of the such strategy is that the incorporation of polyethylene glycol (PEG) as a side chain in a linker contributes to increase hydrophilicity and decrease plasma clearance (29,30). Although the PEGylate linker has been a valid approach to improve the pharmacokinetics properties of ADCs, there are some limitations such as hypersensitivity, non-biodegradablity and accelerated blood clearance.

Currently, polysarcosine (PSR), an intermediate and byproduct of glycine synthesis and degradation, is considered a promising alternative to PEG in terms of biodegradablility and ease of synthesis and versatility (31,32).

While payload masking linkers are impressive methods for ADCs hydrophilicity, hydrophilic payloads are another approach to ADCs stabilisation and enables high DAR. For example, β-D-glucuronyl-monomethylauristatin E (MMAU) is the novel payload which enable high DAR = 8 ADCs with stability and efficacy due to its hydrophilicity (33).

The therapeutic effect will be enhanced by increasing the number of payloads in the ADCs. Most ADCs linkers used so far have linear structures, allowing only a single payload attachment. However, branched linkers enable the loading of two or more payload molecules while maintaining the ADCs’ stability (34,35).

The use of dual payload is another approach. Since in conventional cancer chemotherapy, combining two or more different anticancer agents have enhanced the therapeutic effect, it is expected that loading multiple payloads in ADCs would also enhance the antitumor effect. Several studies have been reported about dual payloads ADCs with antitumor activity, such as MMAE and MMAF (36), MMAE and pyrrolobenzodiazepine (37), MMAF and PNU-159682 (38).

Bispecific antibodies provide another possibility to increase clinical benefit. Their selectivity of binding to two different tumor targets enhances the antitumor effect and reduces the off-target toxicity (39). Several ADCs with bispecific antibodies are currently under development including Integrin/HER2 bispecific ADCs (40), HER2/CD63 bispecific ADC (41) and HER2/prolactin receptor (PRLR) bispecific ADC (42).

Moreover, the combination therapy of ADCs with other antitumor agents, including cytotoxic drugs, antiangiogenic agents, targeted antibodies and ICIs, is currently investigated (43).

## Optimising the safety of ADCs

The toxicity of ADCs varies widely among individuals due to organ functions, comorbidities, pharmacogenomics and so on. Several methods have been considered to optimise the dose of ADCs.

### Antibody modifications

When target antigens are expressed in both tumor cells and normal tissues, on-target adverse events are increased. Probody-drug conjugates (PDC) are antibody prodrugs designed to remain intact in normal tissue by masking antigen-binding regions, while they are activated by protease in the tumor microenvironment. As a result, this approach enables safer binding to target antigens expressed in both cancer and normal cell (44).

First-in-Human studies of CX-2029 and Praluzatamab ravtansine (CX-2009) revealed relevant improvements in the safety profiles. CX-2029 is a PDC comprising an anti-CD71 antibody conjugated to MMAE. CD71 is previously considered as undruggable ADC target because of broad expression in both of tumor and normal tissue. However, the phase I study of CX-2029 revealed the tolerability of targeting therapy for CD71 (45).

Praluzatamab ravtansine (CX-2009) is a PDC consisting of an anti-CD166 antibody and DM4. Although CD166 is also widely expressed in tumor and normal tissues, the results of phase I study of praluzatamab ravtansine showed tolerable safety profiles (46).

In addition, bispecific antibodies, binding to two different antigens, increase, selectivity resulting in the reduction of the off-target toxicity (39).

### Modifying conjugation technology or drug/linker chemistry

#### Tandem linker

One drawback associated with cleavable linkers is the potential to release payloads in plasma circulation before tumor targeting. If the payloads are released systemically prematurely, the efficacy of the remaining circulating ADCs may be reduced, leading to off-target toxicity. Tandem cleavable linkers are novel linkers that incorporate a β-glucuronide moiety and require tandem enzymic cleavage events (47). These linkers reduce payloads release during circulation and off-target toxicity.

#### Silencing the Fc portion of the ADCs

Whereas the Fc domain of the antibody induces immunogenicity, the internalisation of ADCs into non-targeted cells via Fcγ receptors on immune cells may provoke off-target toxicities. By silencing the Fc domain, Fc-mediated off-target cytotoxicity was reduced (48).

#### Novel site specific conjugation technologies

Conventional antibody conjugates have been constructed through cysteine or lysine residue side chains. These approaches were stochastic, non-specific conjugation and generated heterogenous ADCs. To overcome the drawback of non-specific conjugation, intense research to realise site specific conjugation has been conducted. Chemo-enzymatic methods include transglutaminase and glycan-mediated conjugation, and chemical methods include selective reduction of disulfides and N-terminal amine modifications (49).

### Dose-optimisation strategy

Five dose-optimisation strategies are known to minimise toxicity and maximise efficacy. These include body weight-based dose capping, treatment duration capping, dose schedule, response-guided dosing and randomised dose-finding (50).

#### Body weight-based dose-capping

Body weight has been generally considered as significant factor to adjust drug dosage. Body weight-based dose-capping was adapted for EV, ADCs used in urothelial cancer patients (51). The recommended dose of EV was 1.25 mg/kg, but the maximum dose was decided as 125 mg.

#### Treatment duration capping

Limiting the treatment duration is widely adopted when using taxanes or platinum salts to minimise chronic toxicities such as peripheral neuropathy (52,53).

Polatuzumab vedotin, ADCs for diffuse large B cell lymphoma patients, is used up to six cycles. Predicted risk of developing grade ≥ 2 peripheral neuropathy with polatuzumab vedotin increases by ≥50% when eight versus six treatment cycles are administered. These data supported the approval of polatuzumab vedotin for patients with relapsed and/or refractory diffuse large B cell lymphoma at a dose of 1.8 mg/kg every 3 weeks for a maximum of six cycles (54).

#### Dose schedule

Dose frequency is fundamental for the safety profile of a drug. Dose fractionation offers the almost same cumulative dose with a lower peak plasma concentration (Cmax) compared with a single dose and reduce toxicity related to the Cmax. Within the FDA approved ADCs, 4 ADCs (gemtuzumab ozogamicin, inotuzumab ozogamicin, SG and EV) are administered weekly dosing (51,55–57).

#### Response-guided dosing

Response-guided dosing is a flexible strategy to modify the dose of drug based on initial response of individual patient. This method is used in inotuzumab ozogamicin, consisting of anti-CD22 antibody and calicheamicin. The subsequent dose is decreased in patients with complete response after an initial dose of inotuzumab ozogamicin (58).

#### Randomised dose-finding study

Although conventional Phase II/III studies have been arranged to evaluate primary clinical efficacy and safety, these studies often lack data on multiple dose levels. Randomised dose-finding study is conducted to reveal an optimal dose level based on data of multiple dose levels. DESTINY-Lung02 is an example in which the optimal dose of T-DXd (5.4 vs 6.4 mg/kg) for NSCLC was assessed (16). The confirmed ORR was 49.0% (95%CI, 39.0–59.1) and 56.0% (95% CI, 41.3–70.0), median DOR was 16.8 months (95% CI, 6.4- not estimable [NE]) and NE (95% CI, 8.3-NE) with 5.4 and 6.4 mg/kg, respectively. Grade ≥ 3 TEAEs were observed in 38.6 and 58.0% of patients with 5.4 and 6.4 mg/kg, respectively. ILD occurred in 12.9 and 28.0% of patients with 5.4 and 6.4 mg/kg, respectively. Based on this result, the approval dose of T-Dxd was 5.4 mg/kg for NSCLC.

Randomised dose-finding studies might be reasonable for other ADCs and indications, particularly for those in which fatal adverse events like as ILD and/or considerable grade ≥ 3 toxicities are observed either in trials or clinical practice.

### Inverse targeting strategy

The inverse targeting concept is another strategy to reduce off-target toxicity by payload-binding selectivity enhancers (PBSE). PBSE is payload-binding fragments which is co-administered with ADCs to bind unconjugated payload and decrease distribution of payload into non-targeted cells. Preclinical study showed that anti-MMAE antibody fragment and anti-maytansinoid single domain antibody increased the therapeutic window of MMAE-based ADCs and DM4-based ADCs, respectively (59,60).

### Pharmacogenomics

Pharmacogenomics is the term that has been used to mean the study of how a person’s genetic characteristics alter their response to drug. Such genomic technologies are expected to clarify in which patients the toxicity is enhanced (61).

As an example of pharmacogenomics, UGT1A1 polymorphisms is known to cause delayed metabolism of SN-38. SN-38, the active metabolite of irinotecan, is glucuronosylated and excreted by UGT1A1 (62). UGT1A1 has a genetic polymorphism, and patients homozygous or heterozygous for either UGT1A1*6 or UGT11*28 are known to have a delayed metabolism of SN-38 resulting in higher incidence of toxicity.

This correlation was observed as well in the study of ADCs. SG, loading SN-38 as payload, reported that the toxicity was increased in patients with deleterious UGT1A1 polymorphisms (63).

## Conclusions

In summary, we have reviewed the structure and mechanism of ADCs, clinical data of approved ADCs for solid tumors. Based on the ability to deliver novel payloads selectively to targeting cancer cells, they have enabled more effective treatment with fewer side effects. Some ADCs show efficacy in multiple types of solid tumors, which suggests that ADCs may enable cancer treatment based on not only tumor types but also cell surface biomarkers. Further development on ADCs will bring new strategies for cancer treatment.

## Funding

This research received no commercial or financial incentives.

## Conflict of interest statement

Dr Takakura has no conflict of interest. Dr Shimizu has received research funding from AbbVie, Eli Lilly, LOXO Oncology, Novartis, Daiichi-Sankyo, Takeda Oncology, Bristol-Myers Squibb, Eisai, Incyte, AstraZeneca, Pfizer, Chordia Therapeutics, Astellas, Symbio Pharmaceuticals, 3D-Medicine, PharmaMar, Parexel, IQVIA; personal honoraria for lectures, speakers bureaus from Chugai, Taiho, MSD, IQVIA; support for attending meetings and travel from Roche; participation on a Data Safety Monitoring Board or Advisory Board of AbbVie, Chordia Therapeutics, Daiichi-Sankyo, Kyowa Kirin, Chugai; leadership or fiduciary role in other board, society, committee or advocacy group, paid or unpaid of ESMO Targeted Anticancer Therapies (TAT), Asia Pacific Oncology Drug Development Consortium (APODDC), External IRB Member of Phase 1 Trials in Hong Kong, HKSAR, China. Dr Yamamoto has received research funding from AstraZeneca, Chugai Pharma, MSD, Taiho Pharmaceutical, Boehringer Ingelheim, Novartis, AbbVie, Amgen, Asahi Kasei, Janssen, Bristol-Myers Squibb Japan, IQVIA, EPS Corporation, Amgen, A2 Healthcare, Mebix, Ono Pharmaceutical; personal consulting fees from AstraZeneca, Chugai Pharma, MSD, Lilly Japan, Amgen, Novartis, Ono Pharmaceutical; honoraria for speakers bureaus, manuscript writing or educational events from AstraZeneca, Chugai Pharma, MSD, Takeda, Acuuray, AbbVie, Amgen, Ono Pharmaceutical, Guardant Health, Kyorin, Daiichi Sankyo, Taiho Pharmaceutical, Tsumura & Co., TERUMO, Lilly Japan, Boehringer Ingelheim Japan, Novartis, Pfizer, Miyarisan pharmaceutical, Merck biopharma, Janssen; participation on a Data Safety Monitoring Board or Advisory Board of AstraZeneca.
